# Loss of glucocorticoid rhythm induces an osteoporotic phenotype in female mice

**DOI:** 10.1111/acel.13474

**Published:** 2021-09-30

**Authors:** Maaike Schilperoort, Jan Kroon, Sander Kooijman, Annelies E. Smit, Max Gentenaar, Kathrin Mletzko, Felix N. Schmidt, Leo van Ruijven, Björn Busse, Alberto M. Pereira, Natasha M. Appelman‐Dijkstra, Nathalie Bravenboer, Patrick C.N. Rensen, Onno C. Meijer, Elizabeth M. Winter

**Affiliations:** ^1^ Department of Medicine Division of Endocrinology Leiden University Medical Center Leiden The Netherlands; ^2^ Einthoven Laboratory for Experimental Vascular Medicine Leiden The Netherlands; ^3^ Department of Osteology and Biomechanics (IOBM) University Medical Center Hamburg‐Eppendorf Hamburg Germany; ^4^ Department of Functional Anatomy Academic Center for Dentistry Amsterdam (ACTA) Amsterdam The Netherlands; ^5^ Department of Medicine Center for Bone Quality Leiden University Medical Center Leiden The Netherlands; ^6^ Department of Clinical Chemistry Vrije Universiteit Amsterdam, Amsterdam Movement Sciences Amsterdam The Netherlands

**Keywords:** bone health, circadian rhythm, corticosteroids, fracture risk, osteoporosis

## Abstract

Glucocorticoid (GC)‐induced osteoporosis is a widespread health problem that is accompanied with increased fracture risk. Detrimental effects of anti‐inflammatory GC therapy on bone have been ascribed to the excess in GC exposure, but it is unknown whether there is also a role for disruption of the endogenous GC rhythm that is inherent to GC therapy. To investigate this, we implanted female C57Bl/6J mice with slow‐release corticosterone (CORT) pellets to blunt the rhythm in CORT levels without inducing hypercortisolism. Flattening of CORT rhythm reduced cortical and trabecular bone volume and thickness, whilst bone structure was maintained in mice injected with supraphysiologic CORT at the time of their endogenous GC peak. Mechanistically, mice with a flattened CORT rhythm showed disrupted circadian gene expression patterns in bone, along with changes in circulating bone turnover markers indicative of a negative balance in bone remodelling. Indeed, double calcein labelling of bone *in vivo* revealed a reduced bone formation in mice with a flattened CORT rhythm. Collectively, these perturbations in bone turnover and structure decreased bone strength and stiffness, as determined by mechanical testing. In conclusion, we demonstrate for the first time that flattening of the GC rhythm disrupts the circadian clock in bone and results in an osteoporotic phenotype in mice. Our findings indicate that at least part of the fracture risk associated with GC therapy may be the consequence of a disturbed GC rhythm, rather than excess GC exposure alone, and that a dampened GC rhythm may contribute to the age‐related risk of osteoporosis.

## Graphical abstract

Negative effects of glucocorticoid (GC) therapy on bone have been ascribed to the excess in GC exposure, but it is unknown whether disruption of endogenous GC rhythm also plays a role. Here, we show that flattening of the GC rhythm without increasing GC exposure in mice disrupts the circadian clock in bone and results in an osteoporotic phenotype, whilst bone quality is maintained in mice injected with supraphysiologic GCs at the time of their endogenous GC peak. These data indicate that disruption of GC rhythm may underlie age‐related osteoporosis, and could in itself contribute to negative effects of GC therapy on bone.

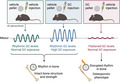

## 1 INTRODUCTION

Glucocorticoids (GCs) are widely used to treat inflammatory and autoimmune disorders on account of their immunosuppressive properties (Rhen & Cidlowski, 2005). Although very effective in reducing inflammation, both short‐ and long‐term GC therapies are associated with serious side effects, amongst which the development of osteoporosis is prominent (Kanis et al., 2004; van Staa, Leufkens, Abenhaim, Zhang, & Cooper, 2000a, 2000b; van Staa et al., 2002; Waljee et al., 2017). Osteoporosis is a progressive bone disease that occurs when there is an imbalance between the activity of bone forming cells, or osteoblasts, and bone resorbing cells, or osteoclasts. When bone resorption exceeds bone formation, this causes an increase in bone fragility and higher fracture risk, which are hallmarks of osteoporosis. Osteoporotic fractures are a frequent cause of morbidity in the elderly and can lead to premature death (Guzon‐Illescas et al., 2019). Whilst sex and age are primary determinants of osteoporosis, GC use is the most common cause of secondary osteoporosis (Chotiyarnwong & McCloskey, 2020).

GCs act on target tissues by binding to both the low‐affinity glucocorticoid receptor (GR) and the high‐affinity mineralocorticoid receptor (MR). Negative effects of pharmacological GC concentrations on bone seem to be predominantly GR‐mediated (Jia et al., 2006; O'Brien et al., 2004; Rauch et al., 2010). High‐dose GCs have been shown to extend the lifespan of osteoclasts in a GR‐dependent manner, by promoting osteoclastogenesis and osteoclast survival (Jia et al., 2006; Weinstein et al., 2002; Yao et al., 2008). GCs also promote the production of receptor activator of NF‐κB ligand (RANKL) by osteoblasts (Swanson et al., 2006), which signals through receptor activator of NF‐κB (RANK) on osteoclasts to further stimulate osteoclast numbers and activity. Prolonged treatment with GCs suppresses differentiation and induces apoptosis of osteoblasts. Although this diminishes activation of osteoclasts through the RANK‐RANKL pathway, it also harmfully reduces bone formation (O'Brien et al., 2004; Rauch et al., 2010). Altogether, these GC effects result in an initial increase in bone resorption, followed by a more progressive reduction in bone formation, thereby inducing bone loss and contributing to osteoporosis development (Henneicke et al., 2014).

It is well‐described that the negative effects of GCs on bone are dose‐dependent (van Staa, Leufkens, et al., 2000a, 2000b). However, the lack of GC rhythm, intrinsic to pharmacological anti‐inflammatory GC administration, could be another potentially contributing factor. In both mice and humans, circulating levels of GCs show a strong diurnal rhythm, with a peak just before onset of the active phase and a trough at the beginning of the resting phase (Chung et al., 2011). This rhythm is regulated by the central circadian clock, also known as the biological clock, located in the suprachiasmatic nucleus (SCN) of the hypothalamus. The SCN modulates the activity of the hypothalamus–pituitary–adrenal (HPA) axis, resulting in the rhythmic release of GCs by the adrenal cortex (Chung et al., 2011). By acting through the GR, GCs regulate circadian rhythm in peripheral tissues, such as adipose tissue and liver (Balsalobre et al., 2000; Kamagata et al., 2017; Oishi et al., 2005; Su et al., 2015). In addition, previous studies revealed a potential role for GCs in rhythmic bone remodelling. GC treatment induces rhythmic gene expression in osteoblasts and osteoclasts *in vitro* (Fujihara et al., 2014; Komoto et al., 2012), and depletion of endogenous GCs by adrenalectomy blunts rhythm in osteoclast‐related gene expression *in vivo* (Fujihara et al., 2014). Altogether this suggests that GC rhythm regulates metabolic activity of bone.

Bone health is also dependent on a regular day–night rhythm (Winter et al., 2021), as reflected in the association between chronic circadian disruption through shift work and osteoporosis in humans (Feskanich et al., 2009; Quevedo & Zuniga, 2010). We have recently demonstrated that shifting light–dark cycles negatively affects bone health in mice (Schilperoort et al., 2020), demonstrating a causal relationship between circadian disruption and bone abnormalities. However, it is currently unknown whether a disrupted GC rhythm, which is also observed with increasing age (Van Cauter et al., 1996) and GC therapy (Spies et al., 2014), could underlie these effects.

In this study we aimed to elucidate the importance of a diurnal rhythm in corticosterone (CORT), the primary GC in mice, for bone quality. We implanted mice with slow‐release CORT pellets to flatten endogenous CORT rhythm without inducing hypercortisolism, and demonstrate that a blunted CORT rhythm results in an osteoporotic phenotype.

## 2 RESULTS

### 2.1 Flattening of corticosterone rhythm reduces lean body mass

**FIGURE 1:**
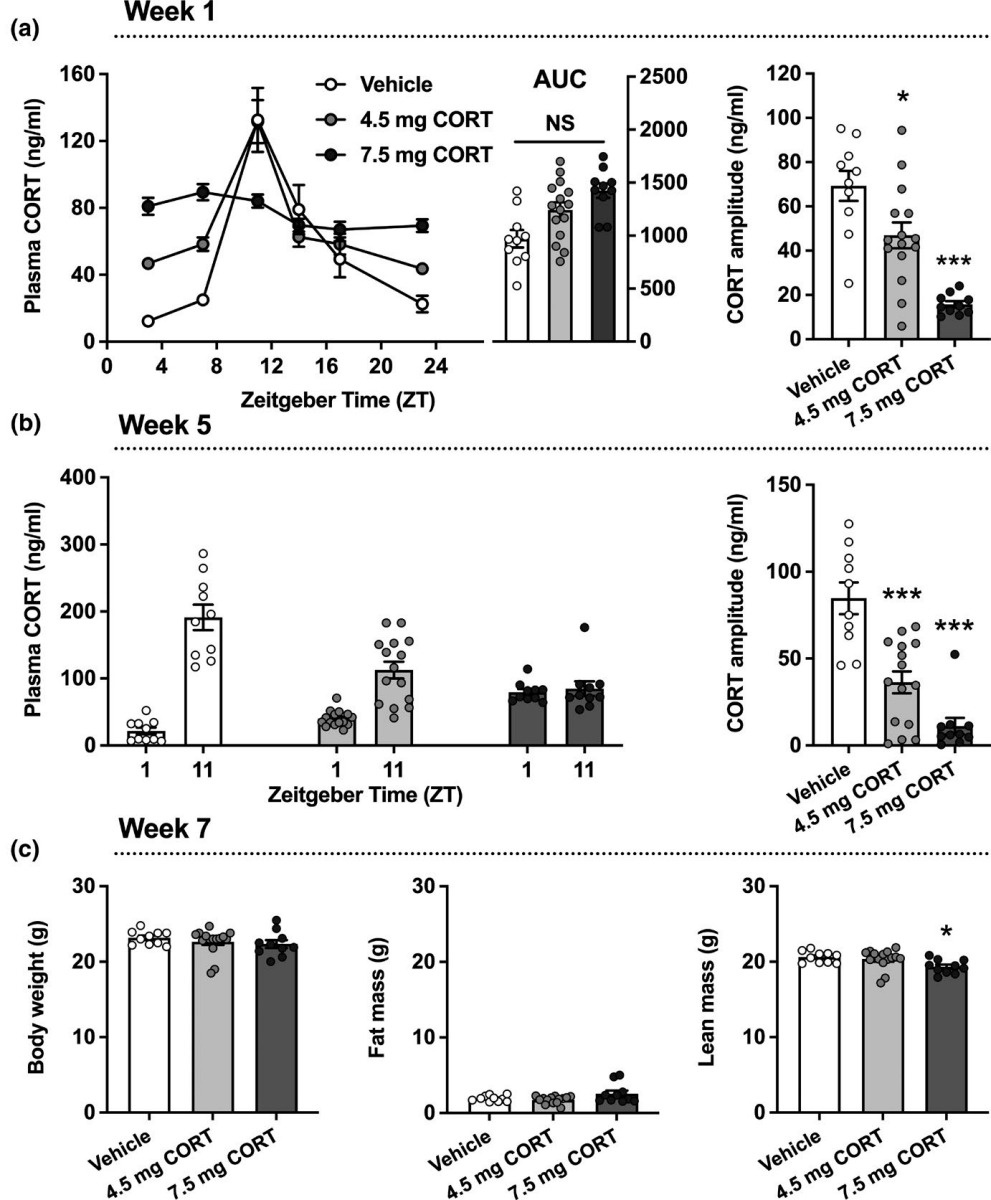
Corticosterone pellets flatten the rhythm in plasma corticosterone and reduce lean body mass. (a) Plasma corticosterone (CORT) levels were measured in mice 1 week after implantation of a vehicle (*n* = 10), 4.5 mg CORT (*n* = 15) or 7.5 mg CORT pellet (*n* = 10), at regular intervals throughout the day and night. An area under the curve (AUC) of all individual plasma corticosterone (CORT) measurements was calculated to determine total CORT exposure (ng/ml*h). For each mouse, a CORT amplitude was calculated by subtracting the lowest value in plasma CORT from the peak value, and dividing this by 2. (b) After 5 weeks of pellet implantation, morning and evening plasma CORT levels were measured and used to calculate the amplitude in CORT rhythm. (c) At endpoint, after 7 weeks of pellet implantation, body weight, fat mass and lean mass were measured. Data represents means ±SEM, including individual data points. Time is denoted as Zeitgeber Time (ZT), where ZT0 = lights on and ZT12 = lights off. *p < 0.05, ***p < 0.001 compared to the vehicle control group, according to one‐way ANOVA with Dunnett's post hoc test. NS, non‐significant

Mice implanted with vehicle pellets showed a strong diurnal rhythm in plasma CORT, with an evening peak of around 150 ng/ml (Figure 1a and 1b). One week after pellet implantation, the 4.5 mg CORT group showed a reduced CORT amplitude (32%, *p* = 0.013), which was due to higher trough levels (Figure 1a). The 7.5 mg CORT group showed an almost completely blunted CORT rhythm (77% in amplitude, *p* < 0.0001), with increased trough values and the absence of a CORT peak at Zeitgeber Time (ZT) 11 (i.e., 1 h before onset of the dark active phase). At endpoint, adrenocorticotropic hormone (ACTH) was dose‐dependently suppressed in mice implanted with CORT pellets (Figure S1a), confirming blunted HPA axis activity. Both the 4.5 and 7.5 mg CORT pellets did not significantly affect the total daily exposure to CORT, as reflected by a similar area under the curve (AUC) of all individual CORT measurements (Figure 1a). In addition, the adrenal weight, a biomarker of GC exposure, was not significantly affected by CORT pellets at endpoint (Figure S1b). In week 5 of the study, the CORT pellets similarly affected CORT rhythm as compared to week 1 (Figure 1b). After 7 weeks of intervention, CORT pellets did not significantly affect total body weight and fat mass, but 7.5 mg CORT pellets did reduce lean body weight (6.2%; *p* = 0.019), indicating loss of muscle and/or bone mass (Figure 1c).

### 2.2 Flattening of corticosterone rhythm results in an osteoporotic phenotype

**FIGURE 2:**
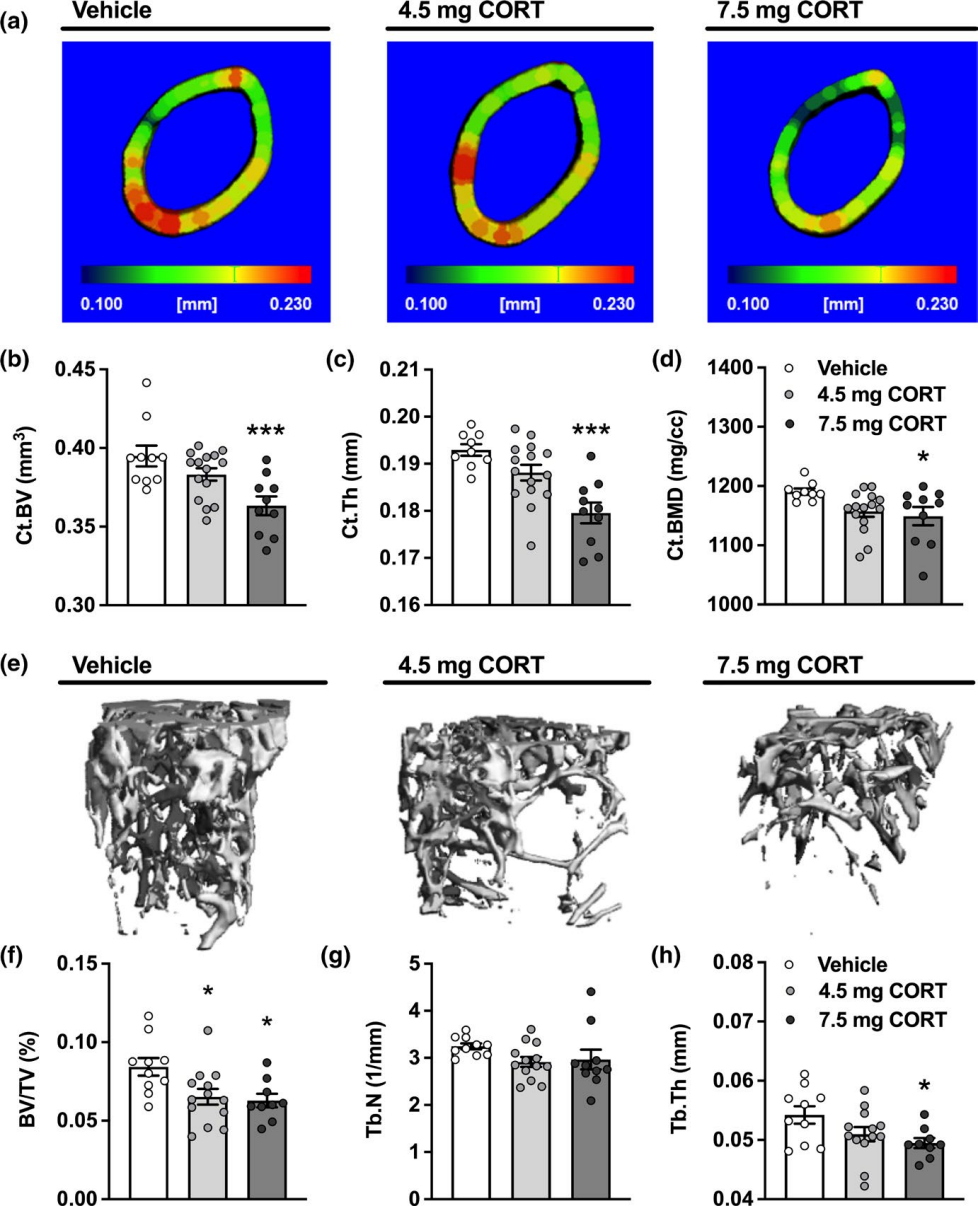
Corticosterone pellets reduce both cortical and trabecular bone volume. Micro‐CT analyses were performed after 7 weeks of pellet intervention. (a) Representative structural images of the cortical bone in all groups, indicating cortical thickness in mm by colour codes. (B)–(D) Micro‐CT analysis was used to assess cortical bone volume (Ct.BV; b), cortical thickness (Ct. Th; c) and cortical bone mineral density (Ct.BMD; d). (e) Representative structural images of the trabecular bone in all groups. (f)–(h) Micro‐CT analysis of the relative trabecular bone volume (BV/TV; f), trabecular number (Tb.N; g) and trabecular thickness (Tb. Th; h). Data represents means ±SEM, including individual data points. *p < 0.05, ***p < 0.001 compared to the vehicle control group, according to one‐way ANOVA with Dunnett's post hoc test

To investigate whether loss of CORT rhythm induces osteoporosis, we evaluated the cortical (Figure 2a) and trabecular (Figure 2e) structure of the femoral bone by micro‐CT analysis. The cortical bone structure of the 4.5 mg CORT group was not significantly different from that of the vehicle group after 7 weeks of intervention, whilst the 7.5 mg CORT group showed a reduced cortical bone volume (8.1%, *p* = 0.0009; Figure 2b), cortical thickness (6.9%, *p* < 0.0001; Figure 2c), and cortical bone mineral density (BMD) (3.4%, *p* = 0.032; Figure 2d). Unlike cortical bone structure, the trabecular bone structure was similarly affected by both 4.5 and 7.5 mg CORT pellets, as both CORT groups showed a strong decrease in relative trabecular bone volume (22.7%, *p* = 0.021 and −25.5%, *p* = 0.017, respectively; Figure 2f). Trabecular number (Figure 2g) and trabecular thickness (Figure 2h) also seemed to be reduced in both CORT groups, although this was only significant for trabecular thickness in the 7.5 mg CORT group (8.7%; *p* = 0.030). We did not observe an effect of CORT pellets on trabecular bone structure of the L4 vertebrae (Figure S2).

**FIGURE 3:**
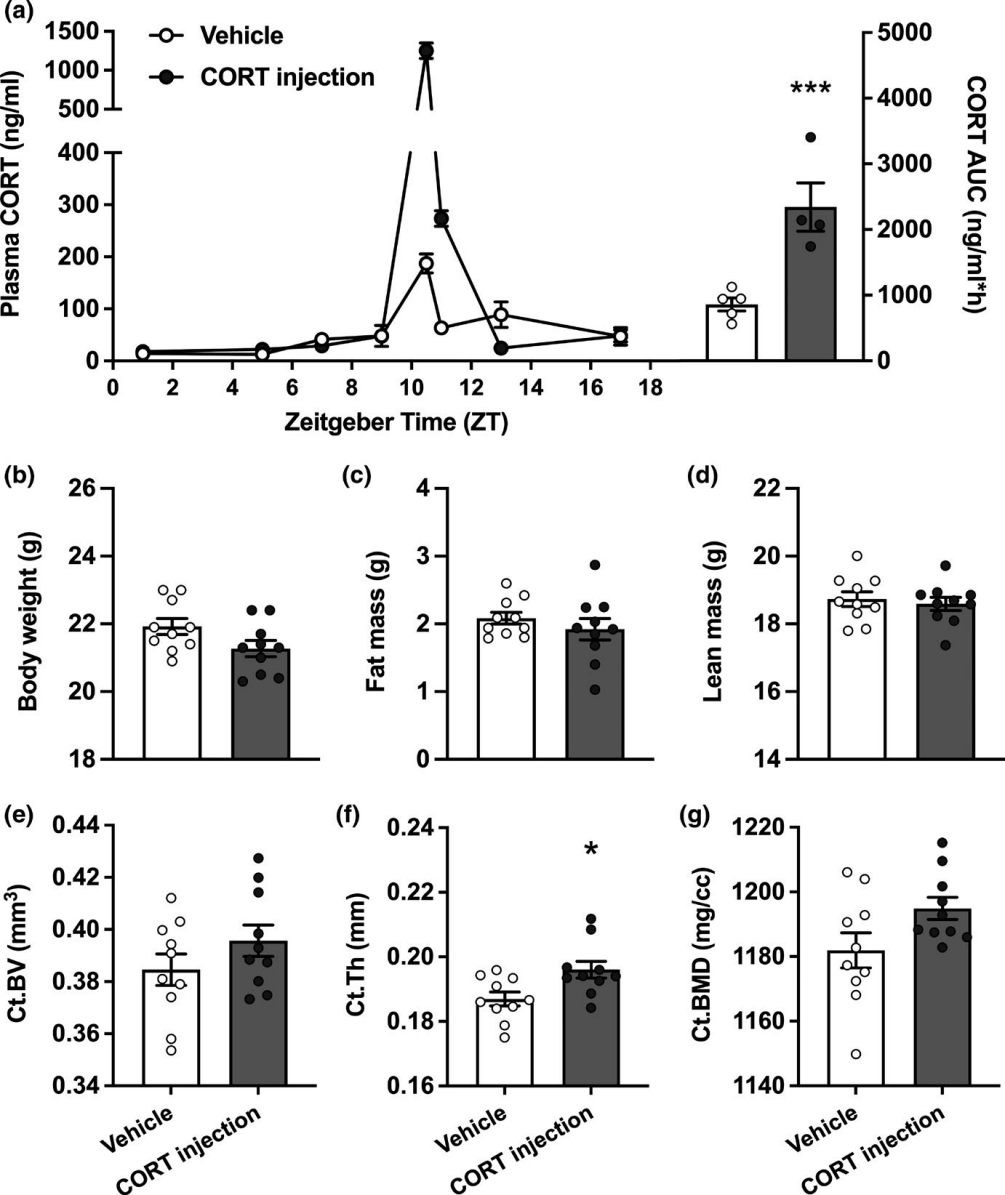
Hypercortisolism with an intact corticosterone rhythm preserves lean mass and cortical bone structure. (a) Plasma corticosterone (CORT) levels were measured in mice 3 days after administration of vehicle (*n* = 10) or 3 mg/kg CORT via daily injection at 17 h (*n* = 10), at regular intervals throughout the day and night (*n* = 5 per group per time point). An area under the curve (AUC) of individual plasma corticosterone (CORT) measurements was calculated to determine total CORT exposure. (b)–(d) At endpoint, after 7 weeks, total body weight (b), fat mass (c) and lean mass (d) were measured. (e)–(g) Micro‐CT analysis was used to assess cortical bone volume (Ct.BV; e), cortical thickness (Ct. Th; f), and cortical bone mineral density (Ct.BMD; g). Data is expressed as means ±SEM, including individual data points. Time is denoted as Zeitgeber Time (ZT), where ZT0 = lights on and ZT12 = lights off. *p < 0.05 compared to the control group, according to unpaired *t*‐test

Of note, these effects on bone structure were not observed in mice injected daily with excess CORT (3 mg/kg) at the time of the endogenous GC peak. Although CORT injections increased the total GC exposure far beyond that of the vehicle group (+173%, *p* < 0.0001; Figure 3a), the CORT‐injected mice showed a completely preserved body composition (Figure 3b–3d) and cortical bone structure (Figure 3e–3g), further supporting that the negative effects of GC (over)exposure on bone are dependent on the time of day.

### 2.3 Corticosterone rhythm is an important regulator of circadian gene expression in bone

**FIGURE 4:**
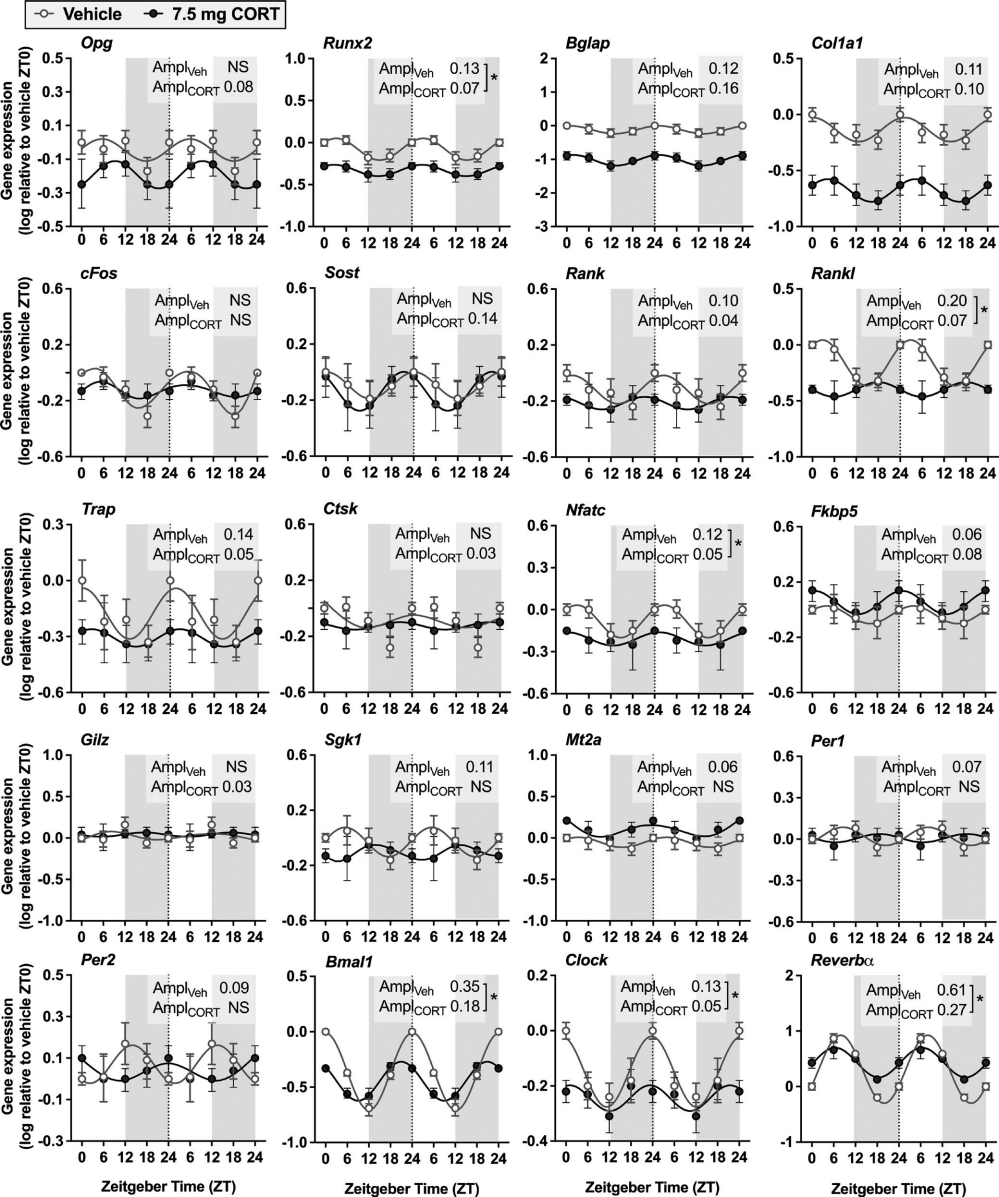
Corticosterone pellets disrupt rhythmic expression of bone‐related genes, glucocorticoid‐response genes, and clock genes. Mice were sacrificed at Zeitgeber time (ZT) 0, ZT6, ZT12, and ZT18 after 2 weeks of pellet intervention to assess circadian rhythmicity of bone‐related genes (*Opg*, *Runx2*, *Blap*, *Col1a1*, *cFos*, *Sost*, *Rank*, *Rankl*, *Trap*, *Ctsk*, *Nfatc*), glucocorticoid‐response genes (*Fkbp5*, *Gilz*, *Sgk1*, *Mt2a*) and clock genes (*Per1*, *Per2*, *Bmal1*, *Clock*, *Reverbα*) in tibiae (*n* = 6/group/timepoint). Data points were log‐transformed and double‐plotted from the dotted lines. Light and grey areas represent the light and dark phase, respectively. Rhythm analyses were performed by fitting a sine wave to the data and evaluating the confidence interval (CI) of the amplitude, as listed in Table S1. Significant amplitudes are shown in the figure. Amplitudes that differ significantly between the two groups are indicated with an asterisk. Data represent means ±SEM. NS, non‐significant or non‐detectable

To investigate potential mechanisms underlying the effect of a flattened CORT rhythm on bone structure, we measured a variety of gene expression markers of bone formation and resorption in the bones collected at ZT11 after 7 weeks of intervention. Although our intervention did not affect the expression of genes involved in bone formation (i.e., *Opg*, *Runx2*, *Blap*, *Col1a1*, *cFos*; Figure S3a) or bone resorption (i.e., *Rank*, *Rankl*, *Trap*, *Ctsk*, *Nfatc*; Figure S3c) at the time of sacrifice, we did observe a decreased expression of markers of GR activation (*Gilz*, 28%; *p* = 0.009; Figure S3b) and the circadian clock (*Per1*, 13%; *p* = 0.028; Figure S3d) in tibia bone samples. We followed up on these findings by evaluating gene expression in tibia throughout the day and night in an additional cohort of mice. After 2 weeks of pellet implantation, we found clear diurnal expression patterns of bone‐related genes, GC‐response genes, and clock genes (Figure 4 and Table S1) in bone of mice implanted with vehicle pellets, which was disrupted in mice that received 7.5 mg CORT pellets. In addition, the expression of *Opg*, *Runx2*, *Bglap* and *Col1a1* was lower in the CORT pellet group as compared to vehicle across all timepoints (Figure 4; *p* < 0.0001 according to two‐way ANOVA).

### 2.4 Flattening of corticosterone rhythm disturbs the balance in bone turnover by favouring bone resorption

**FIGURE 5:**
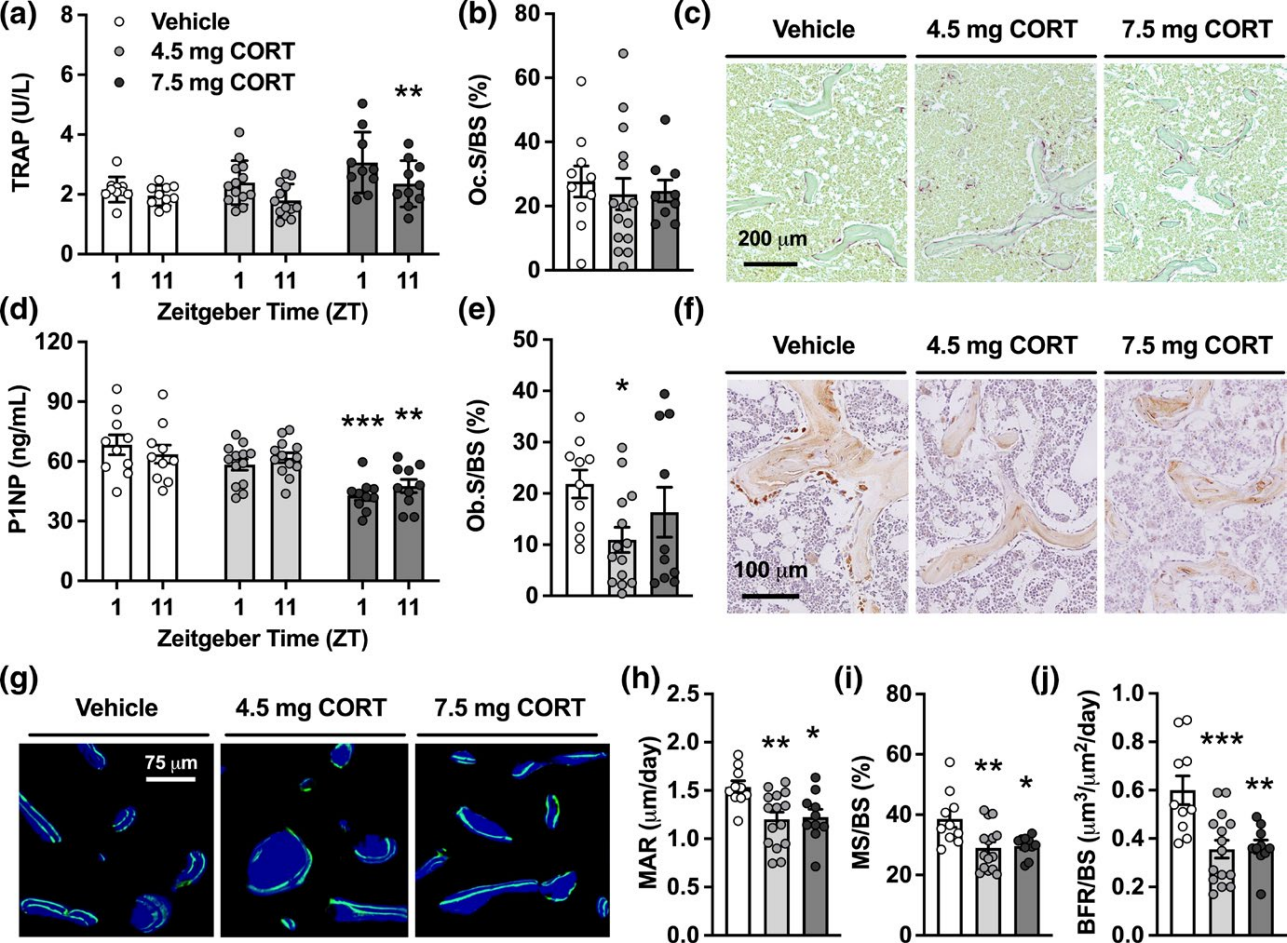
Corticosterone pellets modulate bone turnover markers and reduce bone formation. (a) Plasma levels of tartrate‐resistant acidic phosphatase (TRAP) were evaluated in the morning and evening after 5 weeks of pellet intervention. (b), (c) At endpoint, after 7 weeks of intervention, the trabecular osteoclast surface area (b) was determined from femurs stained for TRAP and counterstained with Light Green (c). (d) Plasma levels of procollagen type 1 amino‐terminal propeptide (P1NP; b) were evaluated in the morning and evening after 5 weeks of pellet intervention. (e, f) After 7 weeks, the trabecular osteoblast surface area (e) was determined from femurs stained for osteocalcin and counterstained with haematoxylin (f). (g) Representative images of femoral trabecular bones double labelled with calcein (green), and counterstained with calcein blue (blue). (h)–(j) Calcein labelling was used to determine the MAR (h), mineralizing surface per bone surface (MS/BS; i) and bone formation rate per bone surface (BFR/BS; j). Data represent means ±SEM, including individual data points. Time is denoted as Zeitgeber Time (ZT), where ZT0 = lights on and ZT12 = lights off. *p < 0.05, **p < 0.01, ***p < 0.001 compared to the vehicle control group, according to two‐way ANOVA (a, d) or one‐way ANOVA with Dunnett's post hoc test (b, e, h–j)

Aside from effects on gene expression in bone, we observed effects of CORT pellets on circulating bone turnover markers and bone histology. In week 5 of the study, the 7.5 mg CORT pellet group demonstrated an increase in plasma levels of the bone resorption marker tartrate‐resistant acidic phosphatase (TRAP) specifically in the morning at ZT1 (+42%; *p* = 0.008; Figure 5a), whilst the osteoclast surface area of trabecular bone was the same in all groups at the end of the study (Figure 5b and 5c). Plasma levels of the bone formation marker procollagen type 1 amino‐terminal propeptide (P1NP) were decreased in the 7.5 mg CORT pellet group after 5 weeks (Figure 5d), in the morning at ZT1 (37%; *p* < 0.0001) as well as the evening at ZT11 (25%; *p* = 0.005). At endpoint, both CORT pellet groups showed a reduction in osteoblast surface area of trabecular bone (Figure 5e and 5f), which was significant for the 4.5 mg CORT pellet group (50%; *p* = 0.046). To further examine effects of a flattened GC rhythm on the circadian profile of bone turnover markers, we measured circulating TRAP and P1NP throughout the day and night in the mice that received a vehicle or CORT pellet for two weeks only. In these mice, levels of TRAP were found consistently higher and levels of P1NP were found consistently lower in the 7.5 mg CORT group as compared to vehicle, without a significant time of day effect (Figure S4). Reduced P1NP levels were also observed in adrenalectomized mice implanted with CORT pellets (Figure S5), ruling out any action of endogenous GCs.

To evaluate whether the reduced P1NP levels reflect a decrease in bone formation, we performed histological analyses of femoral trabecular bone double labelled with calcein *in vivo* (Figure 5g). This revealed a reduction in all indices of bone formation, that is, mineral apposition rate (MAR; Figure 5h), mineralizing surface per bone surface (MS/BS; Figure 5i) and bone formation rate per bone surface (BFR/BS; Figure 5j), in the CORT pellet groups. MAR, MS/BS and BFR/BS were reduced by 22% (*p* = 0.006), 25% (*p* = 0.004) and 41% (*p* = 0.0005), respectively, in the 4.5 mg CORT group, and similarly reduced (by 20% (*p* = 0.018), 23% (*p* = 0.014) and 39% (*p* = 0.002) respectively) in the 7.5 mg CORT group. Collectively, these results suggest that flattening of CORT rhythm disturbs the balance in bone turnover by favouring bone resorption over bone formation.

### 2.5 Flattening of corticosterone rhythm decreases bone strength

**FIGURE 6:**
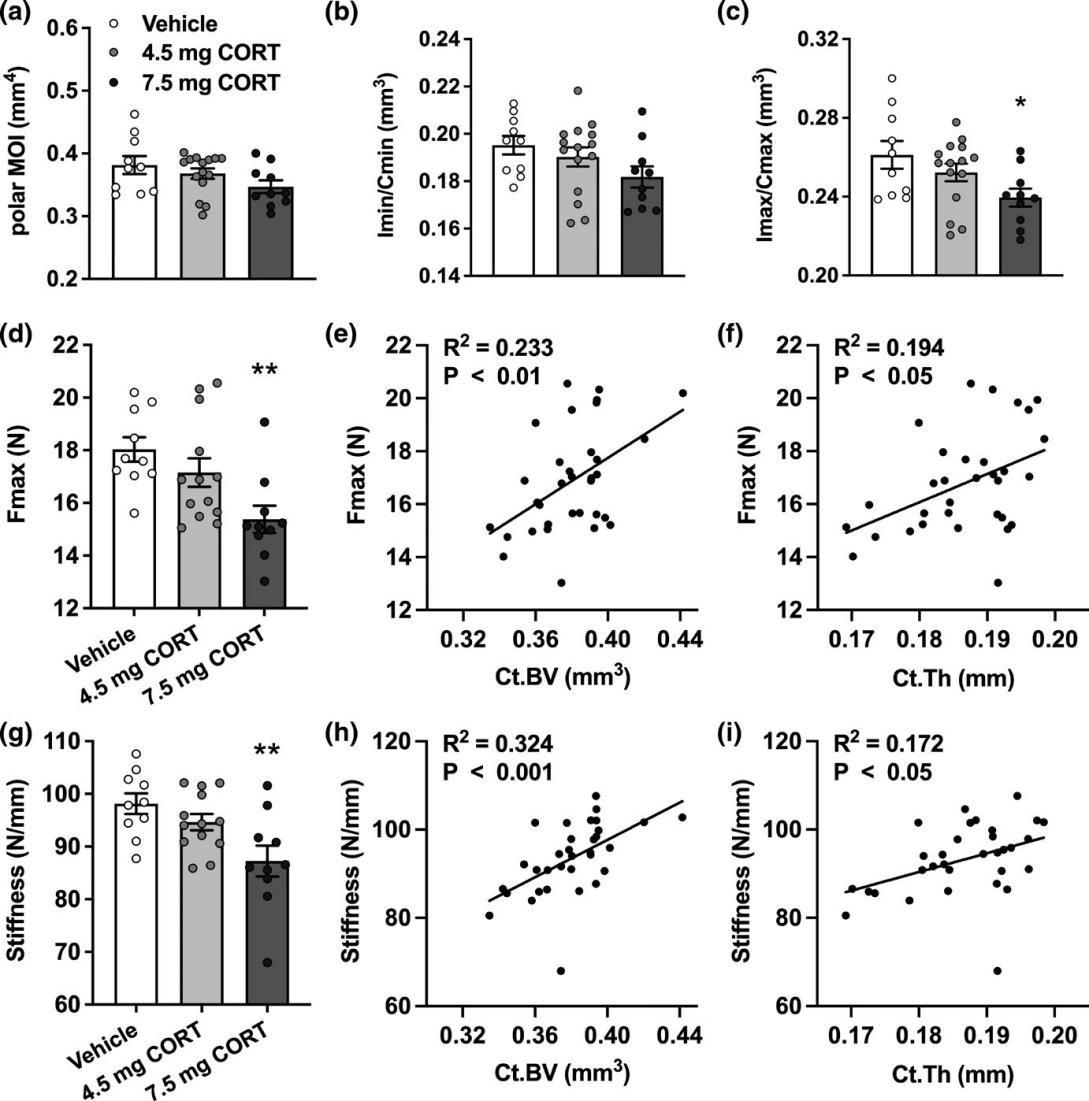
Corticosterone pellets reduce bone strength and stiffness. (a)–(c) Polar moment of inertia (polar MOI; a) as well as two breaking strength parameters, Imin/Cmin (b) and Imax/Cmax (c), were calculated from micro‐CT data. (d) Three‐point bending tests of tibiae were performed to determine the maximum load (Fmax). (e), (f) The relationship between Fmax and femoral cortical bone volume (e) and cortical thickness (f) was evaluated by Pearson correlation analysis. (g) Bone stiffness was evaluated through three‐point bending tests. (h), (i) Bone stiffness was correlated to femoral cortical bone volume (h) and cortical thickness (i). Data represent means ±SEM, including individual data points. **p < 0.01 compared to the vehicle control group, according to one‐way ANOVA with Dunnett's post hoc test

Next, we investigated whether the observed alterations in bone turnover and structure could affect the mechanical properties of bone. To this end, we calculated the polar moment of inertia (MOI), which reflects torsional resistance, as well as two parameters of breaking strength, Imin/Cmin and Imax/Cmax, from the obtained micro‐CT data. The polar MOI was not affected by 4.5 mg CORT pellets, but tended to be lower in the 7.5 mg CORT pellet group (9.1%, *p* = 0.072; Figure 6a). Imin/Cmin also tended to be reduced (6.9%, *p* = 0.080; Figure 6b), whilst Imax/Cmax was significantly reduced (8.3%, *p* = 0.022; Figure 6c), in the 7.5 mg CORT group but not the 4.5 mg CORT pellet group. These calculated parameters point towards a diminished mechanical competence of bone when CORT rhythm is substantially blunted. To substantiate these findings, we performed three‐point bending tests to evaluate the actual cortical bone strength and stiffness of the tibia. Consistent with the fact that cortical bone structure (Figure 2a–2d) and calculated mechanical strength (Figure 6a–6c) were not significantly altered in the 4.5 mg CORT group, we did not find a significant difference in the Fmax, or highest load that the bone can withstand, in this group (Figure 6d). We did observe a substantial decrease in Fmax in the 7.5 mg CORT group (14.7%; *p* = 0.003), indicating a reduced bone strength. Fmax correlated positively with the femoral cortical bone volume (*p* = 0.004; Figure 6e) and cortical thickness (*p* = 0.012; Figure 6f), demonstrating that the variation in bone strength can be partly explained by differences in cortical bone structure. Bone stiffness was also reduced in the 7.5 mg CORT group (11.1%, *p* = 0.003; Figure 6g), and like Fmax correlated positively to both femoral cortical bone volume (*p* = 0.0006; Figure 6h) and cortical thickness (*p* = 0.018; Figure 6i). Taken together, these data imply that a flattened CORT rhythm induces weak and fracture prone bones.

## 3 DISCUSSION

In this study, we evaluated the importance of GC rhythm for bone health. We demonstrate that flattening of the GC rhythm leads to a situation where bone resorption prevails over bone formation, resulting in an osteoporotic phenotype. Our data indicate that disruption of the GC rhythm may underlie the association between shift work and osteoporosis, as well as age‐related osteoporosis, and could in itself contribute to the negative effects of anti‐inflammatory GC therapy on bone.

GC rhythm was flattened by implanting mice with slow‐release pellets containing low doses of CORT. The setup of this experiment is based on the premise that the high‐affinity MR mediates centrally regulated negative feedback to the HPA axis at physiological levels (Dallman et al., 1989; Ratka et al., 1989). Thus, low dose pellets can blunt HPA axis activity without activation of the GR, as previously described by Akana et al. (1992). This intervention has been shown to abolish both diurnal and ultradian CORT variation in rats (Sarabdjitsingh et al., 2010). In our study, we could therefore blunt endogenous GC production and flatten GC rhythm, without substantially increasing the activity of the lower‐affinity GR.

The 4.5 mg CORT dose indeed reduced the activity of the HPA axis as indicated by decreased ACTH levels at endpoint. Although CORT rhythm amplitude was reduced in this group, the negative feedback exerted by the 4.5 mg CORT pellets was not sufficient to abolish the physiological GC peak. The 7.5 mg CORT dose more strongly suppressed ACTH production and thereby effectively flattened the endogenous GC rhythm. Nevertheless, both the 4.5 mg and 7.5 mg CORT doses did not seem to result in GR overactivity, as classic GR target genes (e.g., *Fkbp5*, *Gilz*, *Sgk1*, *Mt2a*) (van Weert et al., 2019) were not upregulated in bone. Also, we did not observe a reduction in the weight of the adrenal gland of the 4.5 and 7.5 mg CORT pellet groups, further supporting that daily GC exposure in these groups was not increased (Akana et al., 1992; Young et al., 1995). Collectively, these results indicate that the negative skeletal effects in both CORT‐treated groups were not the result of an overall excess in GC signalling, but rather attributable to loss of GC rhythm. In with this notion, our analysis of diurnal gene expression suggests that a blunted GC rhythm interferes with healthy bone remodelling by disrupting the molecular clock in bone.

Previous studies have shown that physiological levels of GCs are important for healthy bone remodelling. Rats that lack endogenous GCs as a result of adrenalectomy show a decreased bone mass (Durbridge et al., 1990). This is also observed in osteoblast‐specific GR‐deficient mice (Rauch et al., 2010), and in mice with osteoblast‐specific overexpression of 11‐β‐hydroxysteroid dehydrogenase 2 (11β‐HSD2), an enzyme that inactivates GCs (Sher et al., 2004, 2006). In the current study, we demonstrate that flattening of the GC rhythm results in bone loss, similar to the effects observed with diminished GC levels, whilst overall GC levels were not reduced in our experiment. This suggests that a lack of GCs may affect bone turnover not only via an absence of GC signalling, but also via a lack of rhythmic input. Mice with a blunted CORT rhythm did not show effects on the periodicity of osteoblast‐specific genes but showed a strong reduction in their overall expression, consistent with the osteoporotic phenotype of osteoblast‐specific GR‐deficient mice (Rauch et al., 2010). Although mice with deletion of the GR specifically in osteoclast lineage cells show normal skeletal development (Kim et al., 2006), we found that a flattened CORT rhythm disrupts both the overall expression and the amplitude of osteoclast‐specific genes. The importance of GC rhythm for individual cell types in bone, and their specific contribution to osteoporosis associated with a loss of GC rhythm, warrants further investigation. Furthermore, additional studies are required to evaluate whether GCs at continuous or cycling levels directly act on bone cells, or whether the observed effects are secondary to actions of GCs on other circadian processes such as food anticipation (Kalvisa et al., 2018). Lastly, as we only found an effect of blunted CORT rhythm on trabecular bone of the femur and not of the lumbar vertebrae, future studies should be directed at investigating differential effects of GC rhythm on the appendicular versus the axial skeleton.

Aside from effects of physiological GCs on bone, effects of pharmacological GC administration on bone have often been ascribed to the overall extent of GC exposure. However, not only high but also low dose GC administration induces osteoporosis in humans (McKenzie et al., 2000; Van Staa, Leufkens, et al., 2000, 2000b). In both conditions, physiological GC rhythm is disrupted due to the long biological half‐life of synthetic GCs (i.e., up to 36 h for prednisone and up to 54–72 h for dexamethasone) (Amin et al., 2014; Nicolaides et al., 2000; Rao, 2005), which could contribute to deleterious effects of GCs on bone health. This is supported by our results, showing that flattening of GC rhythm in mice results in an osteoporotic phenotype, as reflected by a reduced cortical and trabecular bone volume and a diminished bone strength, closely resembling the phenotype observed with pharmacologic GC administration in humans.

Whilst mice implanted with 4.5 mg CORT pellets did show a reduced trabecular bone formation and volume, cortical bone volume and bone strength were not significantly affected. Although we cannot exclude a lack of power, bone health could be partly maintained in the 4.5 mg CORT group due to the CORT peak that was still present, as well as the higher rhythm amplitude as compared to the 7.5 mg group. Restoring the amplitude in GC rhythm by reintroducing a daily GC peak could potentially reduce the negative effects of GC administration on bone. However, a natural trough in GC rhythm may be equally important for bone health. Subtle increases in trough levels may not have a large effect on GR occupancy, but could already result in overactivity of the MR. Like the GR, the MR is expressed in bone and has been suggested to play a role in GC‐induced osteoporosis (Beavan et al., 2001; Fumoto et al., 2014). Therefore, in addition to reintroducing a peak in GCs, it may be important to block GC action by administering a GR and/or MR antagonist at the right time of day, to mimic a trough in GC signalling. Alternatively, modified‐release formulations of short‐acting GCs could be implemented to achieve a high dose of GCs at a specific time, whilst still maintaining the natural timing of peak and trough levels. This approach was shown to be effective in reducing morning stiffness in patients with rheumatoid arthritis without adversely impacting endogenous GC rhythm (Buttgereit et al., 2008; Kirwan et al., 2010), although long‐term impact and effectiveness in reducing symptoms lacking circadian nature remains to be studied. In addition, future research is warranted to investigate whether these novel strategies could rescue part of the osteoporotic phenotype associated with GC therapy.

Another way in which our results could be integrated into clinical applications is by promoting healthy ageing. Ageing not only increases the risk of osteoporotic fractures (Sozen et al., 2017), but is also associated with profound changes in the circadian timing system (Hood & Amir, 2017). With increasing age, circadian output of the SCN declines (Nakamura et al., 2011), thereby reducing the amplitude of cortisol rhythm in humans (Van Cauter et al., 1996). Our findings suggest that a dampened GC rhythm may contribute to the age‐related risk of osteoporosis. Restoring the amplitude in cortisol rhythm could thus be an interesting therapeutic strategy to reduce fracture risk in the elderly, and to promote healthy ageing.

In conclusion, disruption of GC rhythm without hypercortisolism induces an osteoporotic phenotype in mice, similar to the phenotype observed with pharmacologic GC administration in humans. It is estimated that ~1% of the adult population receives long‐term GC therapy (Overman et al., 2013), and this proportion increases with age to 2.5% by ≥70 years (van Staa et al., 2000). The fracture risk associated with long‐term GC therapy ranges from 30% to 50% depending on the age of the patient (Angeli et al., 2006). Thus, a substantial number of individuals are suffering from GC‐induced osteoporosis. Our findings indicate that at least part of the deleterious effects of GC therapy on bone could be attributed to a disturbed GC rhythm, rather than an excess in GC exposure alone. Future studies should investigate whether reintroducing a trough and/or peak in GCs at the right time could prevent or reduce GC‐induced osteoporosis and the associated fracture risk.

## 4 EXPERIMENTAL PROCEDURES

### 4.1 Animals

Mice were group housed (*n* = 4/5 per cage) under standard 12 h:12 h light:dark conditions, and were fed chow diet (Special Diet Services) *ad libitum*. All mouse experiments were performed in accordance with the Institute for Laboratory Animal Research Guide for the Care and Use of Laboratory Animals after having received approval from the Central Animal Experiments Committee (‘Centrale Commissie Dierproeven’, the Netherlands).

To examine the effect of a flattened GC rhythm on bone metabolism, twelve‐week‐old female C57Bl/6J mice (Charles River Laboratories) were randomised to receive either vehicle (*n* = 10), 4.5 mg CORT (*n* = 15) or 7.5 mg CORT (*n* = 10) via slow‐release pellets. After 7 weeks, mice were sacrificed around ZT11 by CO_2_ inhalation and perfused for 5 min with ice‐cold PBS through the left ventricle of the heart. Tibiae were collected for gene expression analysis and mechanical testing, and femurs were collected for micro‐CT analysis and evaluation of calcein labelling.

To evaluate effects of a flattened GC rhythm on circadian patterns in gene expression and plasma bone turnover markers, twelve‐week‐old female C57Bl/6J mice (Charles River Laboratories) were implanted with either vehicle or 7.5 mg CORT pellets, and were sacrificed after 2 weeks at ZT0, ZT6, ZT12, and ZT18 (*n* = 7/group/time point). Tibiae were collected for gene expression analysis, and blood was drawn via heart puncture to measure plasma CORT and bone turnover markers.

To evaluate effects of hypercortisolism with a sustained GC rhythm on bone, twelve‐week‐old female C57Bl/6J mice (Charles River Laboratories) were randomised to receive either vehicle injections or 3 mg/kg CORT injections once daily at ZT10 (*n* = 10/group). After 7 weeks, mice were sacrificed around ZT11 by CO_2_ inhalation and perfused for 5 min with ice‐cold PBS through the left ventricle of the heart. Femurs and vertebral columns were collected for micro‐CT analysis.

An experiment with adrenalectomy was performed to exclude effects of endogenous GC production. Twelve‐week‐old male C57Bl/6J mice (Charles River Laboratories) were adrenalectomized and implanted with 7.5 mg CORT pellets or sham‐operated and implanted with vehicle pellets (*n* = 8/group). After one week, blood was drawn at ZT1 and ZT11 to measure plasma CORT and P1NP.

### 4.2 Corticosterone administration—pellets and injections

CORT was administered either via slow‐release pellets or by subcutaneous injection. Slow‐release pellets (1 cm in diameter and 4 mm thick) were made by compressing 4.5 or 7.5 mg CORT (27840, Sigma‐Aldrich) with cholesterol (100 mg in total) by using a TDP 0 Desktop Tablet Press (LFA Machines Oxford). Pellets were subcutaneously implanted through a small incision in the skin of the upper back of the mice. Every two weeks, a new incision was made and pellets were replaced to prevent encapsulation of the pellet and to ensure a continuous release of CORT. Injections were performed daily at 17 h, around the time of the endogenous GC peak. CORT complexed with 2‐hydroxypropyl‐β‐cyclodextrin (C174, Sigma‐Aldrich) was dissolved in saline and subcutaneously injected at a dose of 3 mg/kg/day.

### 4.3 Adrenalectomy

Bilateral adrenalectomy was performed under isoflurane anaesthesia through a dorsal midline skin incision and lateral retroperitoneal incisions, followed by suturing of the wound. To compensate for the loss in adrenal function, all mice were given drinking water containing 0.9% NaCl.

### 4.4 Body weight and body composition measurements

At baseline and endpoint, body weight was measured with a scale and fat and lean mass were determined with an EchoMRI‐100 body composition analyzer (EchoMRI).

### 4.5 Plasma biochemistry

To determine plasma CORT levels at indicated time points, blood was collected from the tail vein into capillaries within 2 min, before CORT levels rise due to stress of animal handling. Plasma CORT concentrations were measured by ELISA according to the manufacturer's protocol (corticosterone EIA, Immunodiagnostics). Additionally, blood was collected to measure plasma concentrations of P1NP and TRAP using enzyme immunoassay kits (IDS) according to manufacturer's instructions. At endpoint, blood was collected via heart puncture to measure plasma concentrations of ACTH using a double antibody RIA kit according to the manufacturer's protocol (MP Biomedicals).

### 4.6 Micro‐CT analysis

Micro‐CT analysis was performed to evaluate structural changes of the bone. Bones were scanned with a microcomputed tomography system (µCT 40; Scanco Medical AG) using 55 kV, 145 µA, 600 ms integration time and a resolution of 10 µm. Image processing included Gaussian filtering and segmentation with σ = 0.8, support 1, threshold 430 mg hydroxyapatite (HA)/cm^3^ for trabecular parameters and 600 mg HA/cm^3^ for cortical parameters, respectively. For femoral trabecular bone, a total of 150 slides (1.5 mm) starting from 0.1 mm distal to the growth plate were analysed. Vertebral trabecular bone was analysed by selecting and scanning the whole L4 lumbar spine vertebrae using the last rib‐bearing vertebrae as a reference point. For femoral cortical bone, 25 slides above and 25 slides below the exact midpoint of the bone were analysed (total 0.5 mm). Trabecular and cortical volumes of interest (VOI) were chosen by visual inspection. The morphometry of cortical and trabecular bone was performed using the calibrated micro‐CT software uct_evaluation v6.5–3 (Scanco Medical AG). Breaking strength was calculated based on morphometry of the femoral cortical bone, and represented as the minimal and maximal values of the polar MOI (Imin and Imax). Maximal radial extents in the directions perpendicular to Imax and Imin were calculated (Cmin and Cmax) and used to determine section moduli (Imax/Cmax, and Imin/Cmin).

### 4.7 RNA isolation, cDNA synthesis and qRT‐PCR

The proximal and distal ends of the tibiae were cut off, bone marrow was flushed out with cold PBS, and tibiae were stored in RNA*later* Stabilization Reagent (Qiagen) before RNA isolation. For RNA isolation, tibiae were mechanically homogenised in TRIzol RNA isolation reagent (Roche Diagnostics) by using the Mikro‐Dismembrator S (B. Braun Biotech International GmbH) in combination with a 5 mm stainless steel bead (Qiagen), and total RNA was isolated according to the manufacturer's instructions (Roche). 100–500 ng of total RNA was reverse‐transcribed using M‐MLV reverse transcriptase (Promega), qRT‐PCR was performed with a SYBR Green Supermix on a CFX96 PCR machine (Bio‐Rad), and cycle threshold (Ct) values of target genes were normalised using Ct values of the housekeeping genes *β*‐*actin and*/*or Gapdh* to obtain delta Ct values.

### 4.8 Histology and immunohistochemistry

Femurs were fixed with 4% phosphate buffered formalin, decalcified for three weeks in 10% EDTA, paraffin embedded and cut into 5 µm sections. Sections were stained with haematoxylin and eosin (HE) following a standard protocol. To visualise osteoclasts, sections were stained for TRAP using an acid phosphatase kit (Sigma‐Aldrich), and counterstained with a Light Green solution. Osteoblasts were stained using a primary anti‐osteocalcin antibody (ALX‐210–333, 1:1000; Enzo Life Sciences) in combination with a goat anti‐rabbit secondary antibody (Dako). Sections were counterstained with haematoxylin. Slides were digitalized with Philips Digital Pathology Solutions (PHILIPS Electronics) for morphological measurement, and both osteoclast and osteoblast surface areas were quantified using TrapHisto open‐source software (van 't Hof, R. J., Rose, L., Bassonga, E., & Daroszewska, A., 2017).

### 4.9 Calcein double labelling

Fluorescent double labelling of bone was applied by injecting mice intraperitoneally with 15 mg/kg calcein (C0875, Sigma‐Aldrich) in 0.2% sodium bicarbonate 8 days and 2 days prior to sacrifice. After sacrifice, femurs were dissected, fixed with formalin, embedded in methyl methacrylate (MMA) and cut into 7 µm sections using a microtome with a tungsten steel D‐profile knife. Bone was counterstained using calcein blue (M1255, Sigma‐Aldrich), and sections were imaged using a Leica DM6B fluorescence microscope (Leica Microsystems). CalceinHisto open‐source software (van 't Hof et al., 2017) was used to determine the MAR, mineralizing surface per bone surface (MS/BS) and BFR/BS of femoral trabecular bone using consistent thresholds and settings for all images, as determined by visual inspection.

### 4.10 Mechanical testing by three‐point bending

Three‐point bending tests of tibiae were performed using a material testing device with a 0 N to 200 N force sensor (Z.2.5/TN1S, Zwick/Roell). After rehydration in saline for two days, the tibiae were placed with the posterior surface facing up on two bearings separated by 7 mm. The distal tibiofibular junction was placed right over one of the bearings for improved reproducibility. Tibiae were loaded perpendicularly to the shaft of the bone between the two bearings with a rounded‐off indenter that was lowered with a displacement rate of 0.01 mm/s. The response to this load was recorded in force‐displacement curves. Maximum load (Fmax) and stiffness were calculated using the testing software (testXpert 10.1, Zwick GmbH & Co).

### 4.11 Statistical analysis

Statistical analysis was performed using GraphPad Prism (version 7.02 for Windows). Means were compared by unpaired *t*‐test, one‐way or two‐way ANOVA followed by post hoc testing as indicated in the figure legends. Pearson correlation analysis was performed to examine potential linear relationships between variables. Differences between groups were considered statistically significant at *p* < 0.05. Rhythm analyses were performed by fitting a sine wave (Y = BaseLine + Amplitude × sin [Frequency × X + PhaseShift]) to the log‐transformed double‐plotted data. Gene expression was considered rhythmic if the 95% confidence interval of the amplitude did not include zero.

## CONFLICTS OF INTEREST

The authors declare no competing interests.

## AUTHOR CONTRIBUTIONS

MS performed experiments, analysed data, and drafted the manuscript together with EMW. JK, SK, AES, and MG performed experiments. KM, FNS and BB performed mechanical tests of the bone, and LvR performed micro‐CT analyses. AMP, NMA, NB, PCNR, OCM, and EMW helped to conceptualise the project and supervised the project. All authors critically reviewed the manuscript.

## Supporting information

Supplementary Material

## Data Availability

The data that support the findings of this study are available from the corresponding author upon reasonable request.
