# Supplementary material for: Loss of glucocorticoid rhythm induces an osteoporotic phenotype in female mice
Source: Aging Cell. 2021 Sep 30;20(10):e13474. doi: 10.1111/acel.13474 (PMC8520718; doi:10.1111/acel.13474)
Supplement: Supplementary file 1 — Supplementary Material [file ACEL-20-e13474-s001.docx]

**Supplemental data**

**Loss of glucocorticoid rhythm induces an osteoporotic phenotype in female mice**

Maaike Schilperoort, Jan Kroon, Sander Kooijman, Annelies E. Smit, Max Gentenaar, Kathrin Mletzko, Felix N. Schmidt, Leo van Ruijven, Björn Busse, Alberto M. Pereira, Natasha M. Appelman-Dijkstra, Nathalie Bravenboer, Patrick C.N. Rensen, Onno C. Meijer, Elizabeth M. Winter


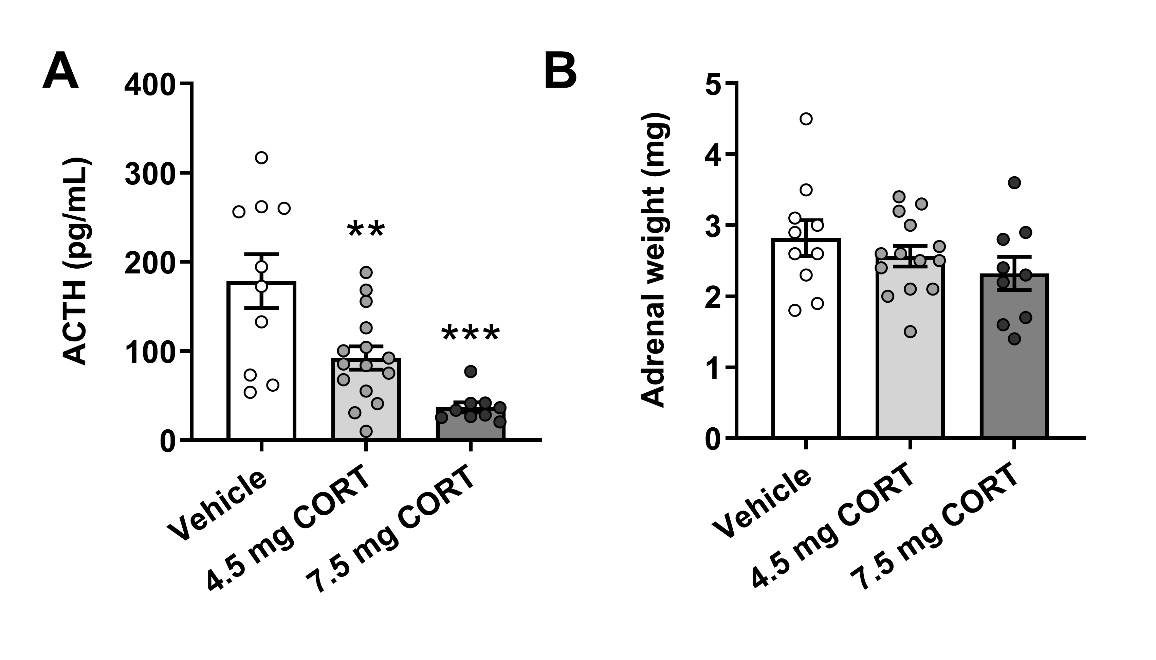


**Supplemental Figure 1 Corticosterone pellets blunt the HPA axis, but do not affect the adrenal weight.** (A) At endpoint, after 7 weeks of vehicle or corticosterone (CORT) pellet implantation, blood was collected to measure plasma concentrations of adrenocorticotropic hormone (ACTH). (B) Weight of the (left) adrenal gland was measured with a scale. Data is expressed as means ± SEM, including individual data points. **P < 0.01, ***P < 0.001 compared to the vehicle control group, according to one-way ANOVA with Dunnett’s post hoc test.

**Supplemental Figure 2 Corticosterone pellets do not affect vertebral trabecular bone.** (A-C) Micro-CT analysis was used to assess relative trabecular bone volume (BV/TV; A), trabecular number (Tb.N; B) and trabecular thickness (Tb.Th; C) of the L4 lumbar spine vertebrae of mice implanted with vehicle or corticosterone (CORT) pellets for 7 weeks (*n* = 10/group). Data is expressed as means ± SEM, including individual data points.


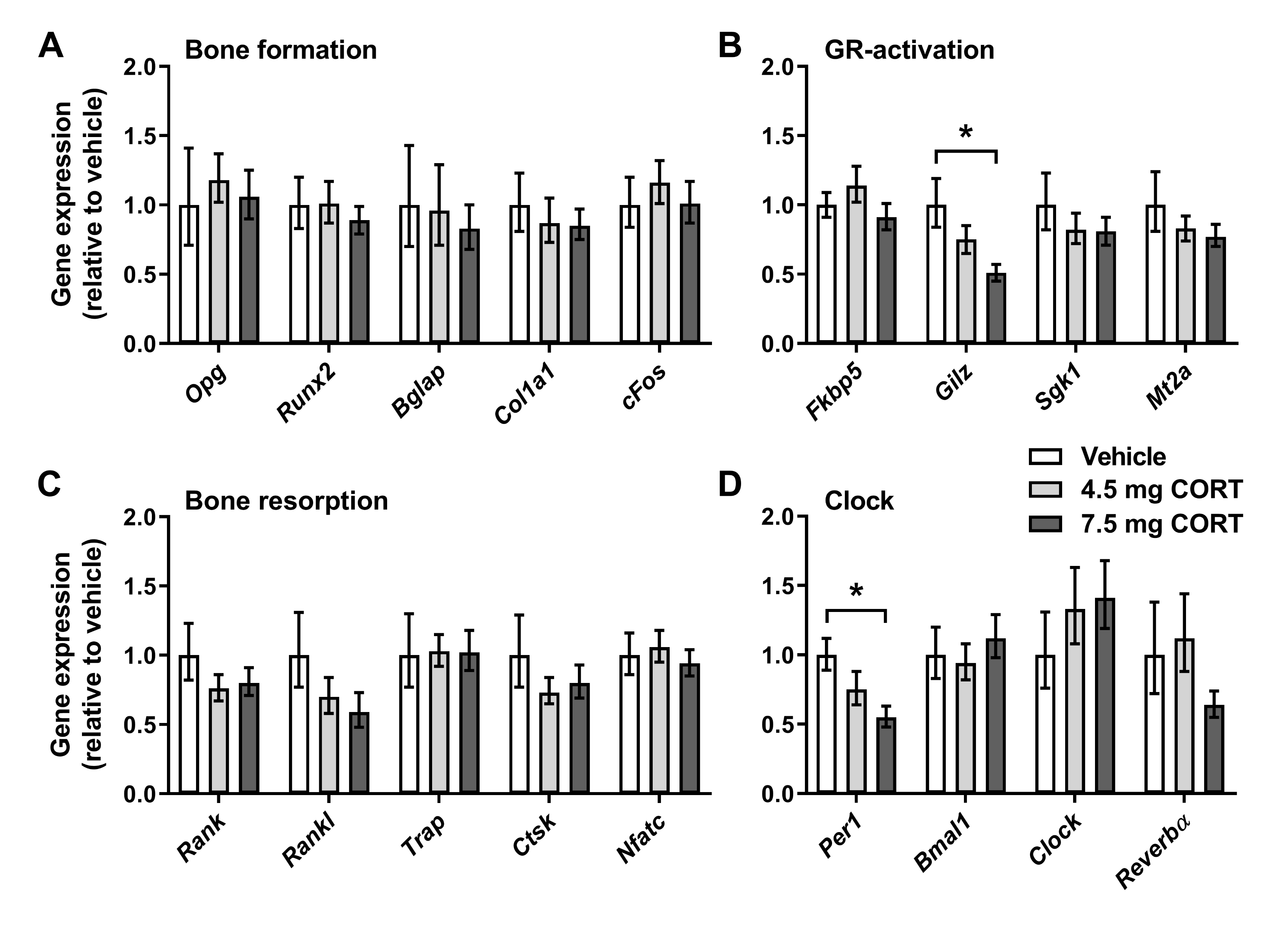


**Supplemental Figure 3 Corticosterone pellets reduce gene expression markers of GR activation and the circadian clock.** (A-D) Relative expression of genes involved in bone formation (A), markers of glucocorticoid receptor (GR) activity (B), genes involved in bone resorption (C) and circadian clock genes (D) was determined in tibia bone samples of mice (*n* = 10-15/group) sacrificed around Zeitgeber Time (ZT) 11 (i.e., 1 h before onset of the dark active phase) after 7 weeks of pellet intervention. Data represents means ± SEM. *P < 0.05 compared to the vehicle control group, according to one-way ANOVA with Dunnett’s post hoc test.

**Supplemental Table 1 Rhythmic gene expression analysis.**

| **Gene** | **Group** | **Amplitude** | **95% Confidence Interval** |
| --- | --- | --- | --- |
| ***Opg*** | Vehicle | 0.07 | -0.03-0.16 |
|  | 7.5 mg CORT | **0.08** | 0.08-0.09 |
| ***Runx2*** | Vehicle | **0.13** | 0.11-0.15 |
|  | 7.5 mg CORT | **0.07** | 0.06-0.07 |
| ***Bglap*** | Vehicle | **0.12** | 0.10-0.14 |
|  | 7.5 mg CORT | **0.16** | 0.13-0.19 |
| ***Col1a1*** | Vehicle | **0.11** | 0.03-0.19 |
|  | 7.5 mg CORT | **0.10** | 0.10-0.11 |
| ***cFos*** | Vehicle | ND | ND |
|  | 7.5 mg CORT | ND | ND |
| ***Sost*** | Vehicle | ND | ND |
|  | 7.5 mg CORT | **0.14** | 0.13-0.14 |
| ***Rank*** | Vehicle | **0.10** | 0.02-0.18 |
|  | 7.5 mg CORT | **0.04** | 0.02-0.06 |
| ***Rankl*** | Vehicle | **0.20** | 0.18-0.23 |
|  | 7.5 mg CORT | **0.07** | 0.06-0.07 |
| ***Trap*** | Vehicle | **0.14** | 0.01-0.27 |
|  | 7.5 mg CORT | **0.05** | 0.04-0.05 |
| ***Ctsk*** | Vehicle | ND | ND |
|  | 7.5 mg CORT | **0.03** | 0.01-0.05 |
| ***Nfatc*** | Vehicle | **0.12** | 0.10-0.13 |
|  | 7.5 mg CORT | **0.05** | 0.01-0.08 |
| ***Fkbp5*** | Vehicle | **0.06** | 0.05-0.08 |
|  | 7.5 mg CORT | **0.08** | 0.07-0.10 |
| ***Gilz*** | Vehicle | ND | ND |
|  | 7.5 mg CORT | **0.03** | 0.02-0.03 |
| ***Sgk1*** | Vehicle | **0.11** | 0.07-0.14 |
|  | 7.5 mg CORT | ND | ND |
| ***Mt2a*** | Vehicle | **0.06** | 0.02-0.10 |
|  | 7.5 mg CORT | ND | ND |
| ***Per1*** | Vehicle | **0.07** | 0.03-0.10 |
|  | 7.5 mg CORT | 0.03 | -0.01-0.07 |
| ***Per2*** | Vehicle | **0.09** | 0.06-0.12 |
|  | 7.5 mg CORT | ND | ND |
| ***Bmal1*** | Vehicle | **0.35** | 0.32-0.37 |
|  | 7.5 mg CORT | **0.18** | 0.16-0.19 |
| ***Clock*** | Vehicle | **0.13** | 0.08-0.18 |
|  | 7.5 mg CORT | **0.05** | 0.01-0.08 |
| ***Reverbα*** | Vehicle | **0.61** | 0.58-0.65 |
|  | 7.5 mg CORT | **0.27** | 0.21-0.33 |

| *Rhythm analyses were performed by fitting a sine wave (Y = BaseLine + Amplitude × sin [Frequency × X + PhaseShift]) to the data shown in Supplemental Fig. 4 and 5, and the corresponding amplitude and 95% confidence interval is listed. Gene expression was considered rhythmic if the 95% confidence interval of the amplitude did not include zero. Significant amplitudes are shown in bold. Genes of which the amplitude is significantly different between the vehicle and CORT groups (non-overlapping confidence intervals) are highlighted. ND: not determinable; a sine wave could not be fitted to the data.*  *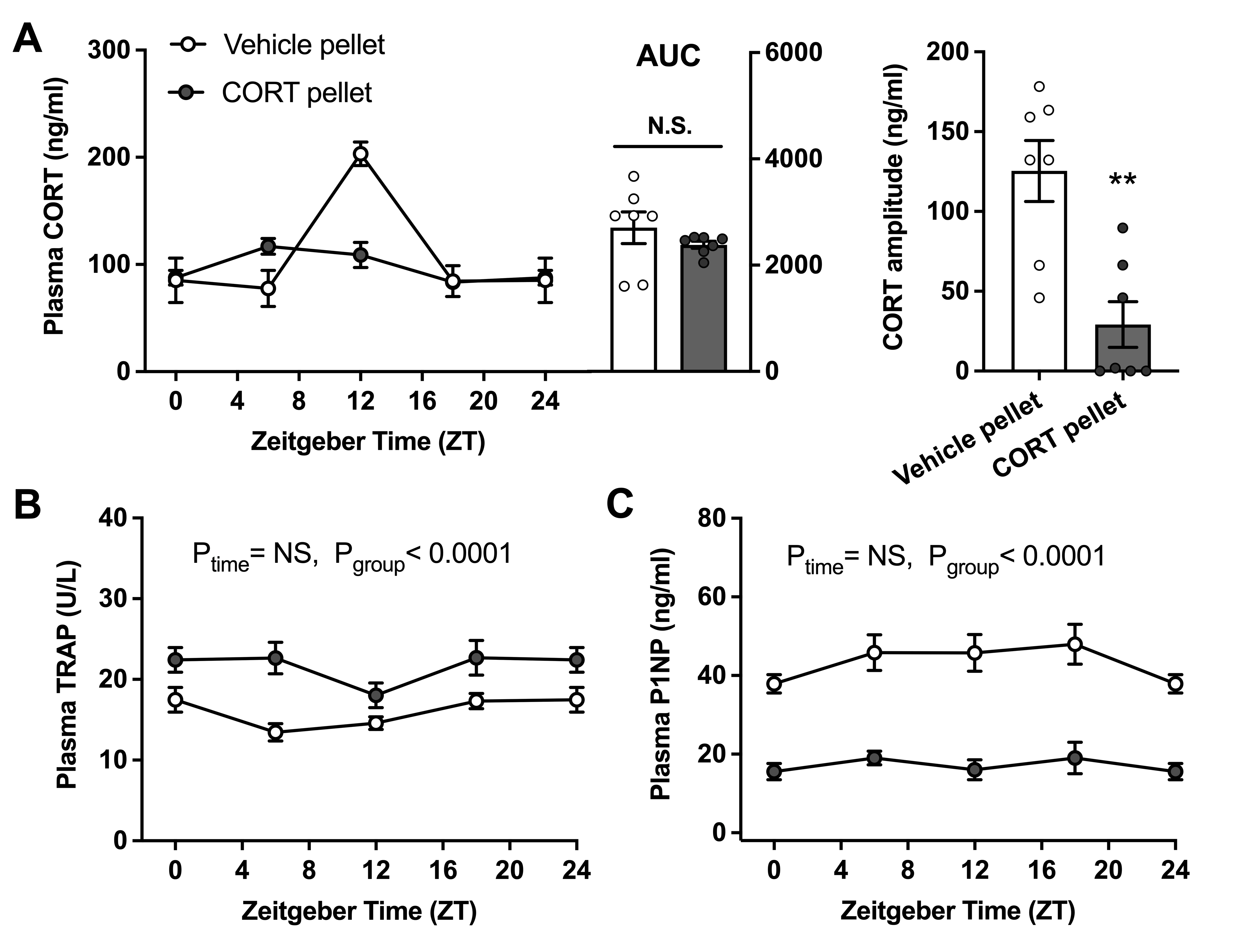* |
| --- |
|  |

**Supplemental Figure 4 The non-rhythmic bone turnover markers TRAP and P1NP are modulated by corticosterone pellets.** (A) Plasma corticosterone (CORT) levels were measured in mice 2 weeks after administration of vehicle or 7.5 mg CORT via slow-releasing pellets (*n* = 7/group/timepoint), at Zeitgeber time (ZT) 0, ZT6, ZT12, and ZT18 (denotation of time in which ZT0 = lights on, and ZT12 = lights off). An area under the curve (AUC) of individual plasma corticosterone (CORT) measurements was calculated to determine total CORT exposure. (B-C) Plasma levels of tartrate-resistant acidic phosphatase (TRAP; B) and procollagen type 1 amino-terminal propeptide (P1NP; C) were measured at the same timepoints. Data is expressed as means ± SEM. **P < 0.01 compared to the indicated control group, according to unpaired T-test. N.S., non-significant. Two-way ANOVA was performed to assess whether group and time are significant sources of variation in TRAP and P1NP, and the corresponding P-values are noted.

**Supplemental Figure 5 Corticosterone pellets flatten the rhythm in plasma corticosterone and reduce P1NP levels in adrenalectomized mice.** (A) Plasma corticosterone (CORT) levels were measured in mice 1 week after implantation of a vehicle pellet combined with sham-operation or implantation of a 7.5 mg CORT pellet combined with adrenalectomy (ADX) (*n* = 8/group), in the morning (ZT1) and evening (ZT11). (B) Plasma levels of procollagen type 1 amino-terminal propeptide (P1NP) were evaluated after 1 week in the morning and evening. Data represents means ± SEM, including individual data points. **P < 0.01, ***P < 0.001 compared to the vehicle control group, according to two-way ANOVA with Sidak’s post hoc test.
